# GlowGrow: Designing an ambient biofeedback system for pregnancy stress management

**DOI:** 10.1371/journal.pone.0320866

**Published:** 2025-06-06

**Authors:** Mengru Xue, Bin Yu, Jiang Wu, Xu Sun, Peidi Fang, Cheng Yao, Fangtian Ying, Shijian Luo, Sheng Zhang

**Affiliations:** 1 Ningbo Innovation Center, Zhejiang University, Ningbo, Zhejiang, China; 2 College of Computer Science and Technology, Zhejiang University, Hangzhou, China; 3 Digital Life Research Group, Faculty of Digital Media and Creative Industries, Amsterdam University of Applied Sciences, Amsterdam, The Netherlands; 4 Faculty of Science and Engineering, The University of Nottingham Ningbo China, Ningbo, China; 5 Nottingham Ningbo China Beacons of Excellence Research and Innovation Institute, Ningbo, China; 6 Obstetrics and Gynecology, Ningbo Yinzhou People’s Hospital, Ningbo, China; Madda Walabu University, ETHIOPIA

## Abstract

Pregnancy can be challenging for women as they experience various physical, psychological, and social changes that can lead to stress and potential mental health concerns. Being neglected in the long-term, sustained stress can increase the likelihood of postpartum depression, which can have significant negative impacts on mothers, families, and society. Therefore, managing stress promptly and maintaining emotional well-being is crucial for pregnant women to give a healthy birth and improve their postpartum life quality. Biofeedback is a secure and effective treatment for anxiety; nevertheless, conventional biofeedback systems often depend on intrusive sensors and require clinician support, thereby restricting their utilization primarily to clinical settings. To address this challenge, in this study, by incorporating biofeedback techniques with wearable sensors, musical displays, and ambient light, we created an immersive biofeedback environment where pregnant women could practice slow-paced resonant breathing to promote relaxation and reduce stress. GlowGrow system has been deployed in a regional hospital’s ante-natal clinic and evaluated by 24 pregnant women regarding its effectiveness and user experience. The results show that GlowGrow, as an effective relaxation intervention, could efficiently guide pregnant women to perform deep breathing and manage physiological stress.

## Introduction

Stress in pregnancy has become a growing concern in our society, which should not be ignored. Prolonged and high-level stress can increase the risk of delivering a premature or low-birthweight baby [1–4]. Furthermore, research suggests that a mother’s prenatal mood can have a lasting impact on her child’s mental health [5, 6]. Awareness of the new sensation of the body leads to physiological hyperventilation [7] associated with pregnancy. The perception of respiratory discomfort implicates breathlessness, commonly found in 70% healthy pregnant women during their daily living activities [8]. These physiological and psychological changes during pregnancy also make pregnant women more vulnerable to various stressors [9]. Therefore, maintaining relaxation and maintaining emotional well-being is crucial for pregnant women’s health as well as birth outcomes [10].

Many studies have proven the emotional and physiological benefits of mindful respiration training in reducing stress and anxiety [11–14]. Instructor-guided relaxation practices are widely applied in obstetrics to help pregnant women cope with stress and improve health outcomes during pregnancy and the postpartum period [15].In addition, as a computer-assisted relaxation technique, Biofeedback and Bio-feedforward systems have been developed to facilitate deep breathing and relaxation training [10, 16–18]. Biofeedback techniques have been shown to be effective in helping people cope better with stress in their daily lives. It has been increasingly accepted and used to treat stress-related diseases during pregnancy [16, 19–22].

Although Biofeedback and Bio-feedforward techniques have the potential to benefit individuals in managing stress, current systems have remained primarily in clinical settings and require support from doctors and wearable sensors. In addition, traditional Biofeedback systems typically feature on-screen displays designed for therapists and clinical users, while non-functional requirements, such as usability, user experience, and engagement, have often been overlooked. This has hindered Biofeedback from becoming a practical stress-coping and relaxation tool for everyday use. Few Bio-feedforward and Biofeedback systems have been tailored to meet the specific needs of pregnant women.

In this study, we developed an ambient Biofeedback system ’GlowGrow’ that can measure users’ heart rate variability and respiration and provide users with multi-modal biofeedback to assist relaxation exercises. The system consists of unobtrusive bio-sensing wearables, a Biofeedback and Bio-feedforward program, and an ambient user interface that combines nature sound, sedative music, and ambient lighting. The GlowGrow ambient interface offers off-screen deep breathing guidance for pregnant women and incorporates environmental audio and lighting stimuli to create an immersive relaxation experience. The system was evaluated with 24 pregnant women regarding its effectiveness and user experience. The results offer fresh perspectives for the design of customized wearable biosensors for pregnant women, along with potential pathways for future development and practical references for creating a biofeedback-driven environment that promotes relaxation and stress management. This study focused on the following research questions (RQs):

- RQ1: What essential design considerations should be taken into account when creating biosensing wearables and ambient user interfaces tailored specifically for pregnant women?
- RQ2: How can light and nature sounds be effectively leveraged in the design of a biofeedback ambient user interface?
- RQ3: How effective is the ambient biofeedback system in reducing stress and inducing relaxation?

In the remainder of the paper, we present the findings of user research using a participatory design approach working together with two families of pregnant women, three obstetricians, two obstetric nurses, and twelve designers to understand the needs and expectations of pregnant women for relaxation training and co-create the potential wearable sensors and ambient biofeedback displays (to answer RQ1). Next, we present the implementation of the GlowGrow system, which integrates wearable HRV and respiration sensors and corresponding Biofeedback and Bio-feedforward algorithms for slow-paced breathing guidance in relaxation exercises (to answer RQ2). Lastly, we discuss the final findings of the user study that evaluated the system in terms of its usability and user experience (to answer RQ3).

## Related work

### Stress during pregnancy

Pregnancy is a time of joy, as well as stress. Researchers found that one in seven pregnant women in developed countries experiences perinatal depression during pregnancy or in the first year after delivery [23, 24]. It is one of the most common mood disorders that occurs twice as often in women as in men, and the onset of depression reaches its peak during a woman’s reproductive years [25]. During pregnancy, a woman goes through many changes physiologically and psychologically. Several factors predispose to depression [5, 26], for instance, economic deprivation, previous abortion experiences, insufficient social support, obsessive worries on pregnancy outcomes and fetal safety [2, 27–29].

Stress during pregnancy can affect various aspects of a pregnant woman’s life, including sleep, energy level, appetite, and libido [30]. Additionally, prolonged stress can have a significant impact on the health of the unborn child, increasing the risk of miscarriage, premature birth, and low birth weight [4, 31]. High levels of norepinephrine and cortisol decrease blood flow into the uterus, which causes problems for both the pregnant women and the fetus [32]. Recent work found that depression during pregnancy also leads to placenta epigenetic changes, which could have implications for the long-term mental health of the child [6, 33]. Numerous symptoms of depression closely resemble the mental and physical changes that occur during pregnancy. This similarity often leads to the dismissal of perinatal depression [34, 35]. When pregnant women experience stress and tension leading up to labor, their contractions can intensify in perceived pain and become less effective. Hence, it is essential to care for the mental well-being of pregnant women, alleviating their stress to ensure the general health of both the mother and the baby [5].

### Pregnancy stress measurement

Stress is typically assessed through bio-sensors or self-report surveys. A growing number of stressors can impact the autonomic nervous system (ANS) activities in pregnant women, potentially causing an imbalance. The dysfunction of the ANS can be observed and assessed using various biomarkers [36, 37] such as heart rate variability (HRV) [38–40], galvanic skin response (GSR) [41], heart rate, blood pressure, etc. Among all these measures, time-domain HRV indexes (e.g., SDNN, RMSSD, AVNN, and pNN20) and frequency-domain HRV indexes (e.g., LF, HF, LF/HF) [42] are widely used and considered reliable HRV parameters for quantifying physiological stress. On the other hand, numerous self-reported stress scales, such as the State-Trait Anxiety Inventory (STAI) [43] and the Relaxation Rating Scale (RRS) [44], have been created to evaluate subjective stress and anxiety. In the field of obstetrics and gynecology, the Pregnancy Stress Rating Scale [45] and the Edinburgh Postnatal Depression Scale (EPDS) [46] are frequently employed to comprehend the specific factors influencing stress in pregnant women. These self-report surveys can aid in early-stage screening for the risk of depression in expectant mothers [47]. Nonetheless, given the subjective and delayed nature of self-reported data collection, it is crucial to incorporate subjective assessment with physiological bio-markers for a more comprehensive and accurate measurement of pregnancy stress.

### Stress-coping and relaxation techniques for pregnant women

Various interventions, characterized by being low-risk, efficient, easy-to-administer, and non-pharmacological, are developed to support pregnant women in stress management and relaxation. Mindfulness [48] and expressive writing [9] train pregnant women to develop the skill of paying attention to their sensations and emotions [49] and overcoming maternal distress, depression, and anxiety [50]. Slow-paced respiration techniques, such as diaphragmatic breathing exercises [51], and Lamaze breathing [52], are extensively employed to alleviate pregnancy stress and prepare for the upcoming childbirth. Slow-paced deep breathing can increase fetal brain oxygen saturation and also enable pregnant women to focus on controlling their breathing during labor and redirecting the perception of pain. Deep breathing can also enhance the calmness of pregnant women, potentially expediting the labor process and facilitating a smoother childbirth experience [8]. The Bio-feedforward technique is extensively used to guide users in breathing regulation. Specifically, it provides users a preset fixed deep breathing guide signal, via visual or audio displays [16, 53], which users follow to engage in deep breathing relaxation exercises.

Beyond Bio-feedforward which solely provides a deep breathing guide, Biofeedback techniques, when coupled with biosensors, are designed to bring unconscious physiological processes under conscious control. This enables users to gain awareness of and learn to self-regulate their internal states through external cues. Biofeedback is proven to be one of the promising interventions for alleviating stress in pregnant women [9, 54]. HRV Biofeedback training is practically effective in regulating users’ autonomic nervous activities [18, 55–57] for stress management. As an example, metronomic respiration training enabled by biofeedback successfully reduced perceived stress and anxiety in pregnant women [58]. HRV biofeedback is the most commonly used biofeedback technique for stress management [103]. HRV biofeedback systems utilize ECG (electrocardiogram) or photoplethysmogram (PPG) sensors to capture users’ heartbeat data and HRV indexes for feedback. Compared to ECG sensors, PPG sensors are less intrusive and more comfortable to wear, making them widely utilized in various daily stress management devices like *emWave* [59, 60], *Wild Divine* [61, 62] and *StressEraser* [63, 64]. However, PPG sensors often require a finger or ear clip for attachment to the skin, impeding the comfort of wearing.

Respiration biofeedback systems can also assist users in managing stress through deep breathing practice. Breathing data is typically measured by a belt-type stretch sensor attached to a user’s thorax or abdomen [65–68]. Additionally, measurement can also be achieved through non-contact sensing techniques, such as signal strength in wireless networks [69], a Doppler multi-radar system [70], or a microwave sensor [71]. These noncontact sensing techniques increase the comfort level, but their accuracy is prone to environmental noises. Furthermore, galvanic skin response (GSR) biofeedback is employed to raise users’ awareness of their arousal levels and help them manage cognitive load [72] and acute stress [73]. EMG biofeedback systems measure specific muscle activities, such as the frequencies and amplitude of the EMG signal of the trapezius muscle, as indicators of mental stress [74, 75]. These systems assist users in relaxing muscles and consequently alleviating stress.

### New HCI design for biofeedback

Recently, there has been a surge in the development of computer-assisted devices and systems aimed at supporting individuals in stress management and relaxation exercises [16, 19, 21, 76]. Numerous emerging Human-Computer Interaction (HCI) technologies have been incorporated into Biofeedback systems to enhance their usability and user experiences. Beyond the traditional on-screen graphic interface, biofeedback information is presented through various user interfaces, including musical [77–79], lighting [76, 80], shape-changing [81], tangible [82], and haptic [83] interfaces. Unlike clinical biofeedback interfaces, which prioritize accuracy and timeliness, these new interfaces are designed to enhance the intuitiveness of biofeedback information, making biofeedback systems more user-friendly.

In the context of stress management and relaxation, integrating ambient interfaces, such as lighting and musical interfaces, into biofeedback systems for information display can be particularly well-suited. Colored light therapy [84] has been increasingly applied to facilitate stress relief in psychological disorders [85]. Nature sounds, such as birds, wind, and rainfall, can foster the experience of calmness and relaxation and are thus widely utilized for stress reduction [86], sleep disorder treatment [87], and mood boost [88, 89]. Being used in ambient displays, as the ’everyday sounds’ around us, nature sounds can be understood intuitively and create a ’calm’ sonic environment, which can engage the periphery of our attention to grab the presented information. The combination of ambient light and nature sounds can create an immersive user experience for relaxation training [22].

The mentioned relaxation techniques and biofeedback interventions have the potential to alleviate symptoms of maternal depression and anxiety. This is likely to be beneficial for both mothers’ mental health and birth outcomes [9]. However, few biofeedback systems have been specifically designed and developed to address the stress management needs of pregnant women in their daily lives [10]. Additionally, to the best of our knowledge, there is currently no research comparing the effectiveness of stress management and user experiences among pregnant women using Biofeedback versus Bio-feedforward techniques. Hence, in this study, utilizing the developed GlowGrow stress reduction system, we delved deeper into examining the potential pros and cons of employing Biofeedback and Bio-feedforward for alleviating pregnancy-related stress.

## User research

### Participants

To comprehend pregnant women’s specific needs and preferences about biosensors and biofeedback display, a series of co-creation sessions were conducted involving two pregnant women, their husbands, one obstetrician, four obstetric nurses, and twelve design professionals, including four user experience designers, four industrial designers, and four HCI designers. To ensure that the recruited pregnant women have a comprehensive experience of pregnancy, both selected participants were in the late stages of pregnancy, at 37 weeks and 36 weeks, respectively. The obstetrician and obstetric nurses (5 females) had over five years of experience in providing obstetric care. In addition, 12 design professionals (6 female, 6 male) were selected from various design institutes. Their length of experience working in design ranged from more than 10 years (4), 5-10 years (2), to 3-5 years (6). The co-creation sessions were organized into two groups. Each group consisted of one pregnant woman, her husband, two obstetric nurses, and six designers. The obstetrician participated in both groups’ sessions. The co-creation sessions were carried out at a regional hospital’s antenatal clinic, where the initial prototype GlowGrow system was implemented.

### Procedure

**Fig 1:**
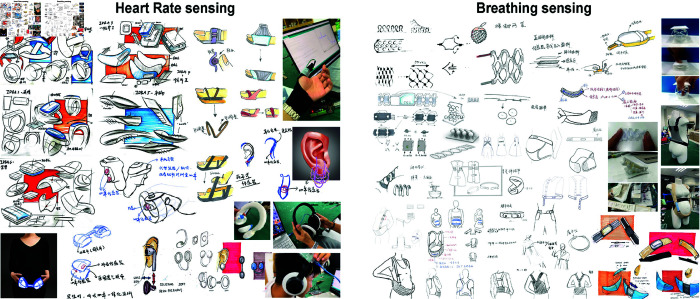
The usage scenario and design concepts of wearable heart rate and breathing sensors generated by the 12 design professionals in the initial stage of user research.

This user research (from April 7, 2023, to May 10, 2023) comprises multiple stages. During the initial stage, the twelve designers conducted product research on wearable bio-sensing products and generated a set of preliminary concepts (see Fig 1). In the second stage, the designers conducted in-depth interviews with each pregnant woman to understand their daily routine, identify the common stressors they frequently experienced, and explore the approaches they often used to cope with stress. In the third stage, pregnant women, obstetricians, and obstetric nurses were all invited to experience the GlowGrow prototype system. This was intended to provide them with a comprehensive understanding of the functionality of the Biofeedback system. Building on this experience, the first co-creation session was carried out to generate ideas for a comfortable and robust method of wearing the heart rate and breathing sensors. In the 30-minute session, the participants discussed the preliminary concepts provided by the design professionals (Fig 1). Subsequently, they drew a list of ideas on paper and shared their ideas within the group. Next, participants engaged in a discussion, highlighting aspects they appreciated about each idea and offering suggestions for potential improvements.

The second co-creation session centered on crafting an ambient biofeedback interface. Using the prototype system, participants experienced the changing ambient light with various color themes ranging from purple and blue to green. They were also immersed in a diverse nature soundscape consisting of various elements, including birdsongs, wind, river sounds, sea waves, and more. Then all participants were also invited to share their experience with changing light and soundscape. They worked together with the designers to create or improve the concepts for the biofeedback display using ambient elements such as light, nature sounds, and music.

### Design considerations

Drawing from the outcomes of the co-creation sessions, we concluded the design concepts for wearable biosensor solutions. Regarding the breathing sensor, the co-created concept is to integrate the breathing belt sensor with the maternity belt, as shown in Fig 4. The breathing belt sensor comprises a fabric textile sensor with inherent elasticity. The resistance of the fabric sensor changes linearly according to the strain it undergoes. Therefore, it is feasible to embed the breathing sensor into the upper fixed strap of the maternity belt to sense pregnant women’s breathing patterns through belly movement. The pregnant women and obstetricians were in favor of this concept, as the maternity belt is a common everyday garment to alleviate discomfort for expecting mothers while facilitating their participation in daily activities. Regarding the heart rate sensor, instead of using a clip to fix the sensor on the fingertip, the participants created new concepts of integrating the sensor into a wearable band to reduce the data noise caused by motion artifacts. As shown in Fig 3, the wearable band could help to fix the sensor on the skin surface and improve the robustness of sensing.

Regarding the ambient system interface, all participants recommended utilizing changes in ambient light brightness to represent breathing patterns. They also suggested adding more light sources (floor lamps and portable lamps) to create a more immersive experience for the users. The pregnant women and obstetricians favored the calming colors of purple and blue to create a more relaxed atmosphere. When it came to the audio interface, the obstetricians suggested using natural sounds that are familiar to the user. They also suggested controlling the number of elements in the created soundscape to prevent noise and maintain a serene sound environment. One obstetrician cautioned that we should reduce the sound of rain because *”the rustling sound is harsh and restless when it becomes loud”.* In addition, the pregnant women mentioned adding relaxing music, supplementing nature soundscape.

Regarding the system’s usage, three obstetricians pointed out that regular, slow-paced breathing practices with biofeedback could assist pregnant women in mitigating the negative effects of stress throughout the entire pregnancy period. Additionally, it could enhance deep breathing skills beneficial during the birthing process. For instance, one obstetrician said, *”Deep breathing can release the pain and make pregnant women relaxed. The more relaxed the mom became, the more smoothly the delivery”.* Another obstetrician stated, *”You can imagine that when the mom is nervous, her whole body is stressed. It can’t be easy for the delivery...Only when she relaxes enough, the cervix will open for the delivery”.* These findings in the user research study guided us in the ideation and iteration of the design of the wearable solutions, user interface, and bio-feedback system. The following sections will delve into the final design and implementation of the product.

## System design and implementation

**Fig 2:**
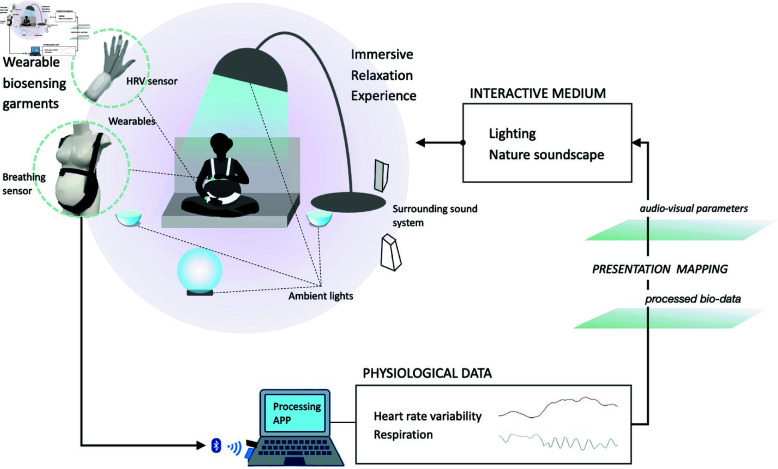
GlowGrow system framework.

In this study, we developed an ambient biofeedback system named GlowGrow. Different from a traditional biofeedback system, it takes an ‘ambient’ form beyond a graphic user interface. This design aims to enable pregnant women to practice slow-paced deep breathing and enhance relaxation skills with greater comfort and fewer restrictions. To achieve this goal, in GlowGrow, we integrated heart rate and breathing sensors into a hand band and a maternity belt to comfortably measure pregnant women’s physiological data as the system input. We leveraged the ambient mediums of light and soundscape in the GlowGrow user interface to present Biofeedback and Bio-feedforward information as the system output. The ambient light and soundscape change with the user’s breathing, also providing the user with an immersive experience for relaxation. As shown in Fig 2, the system of GlowGrow system comprises three components: wearable biosensors serving as the system input, a PC program processing heart rate and breathing data and generating Biofeedback and Bio-feedforward information as the system output, and an ambient user interface that maps the output information to the changes in lighting and sound elements. We will elaborate on each part in the following sections.

### Wearable bio-sensors

**Fig 3:**
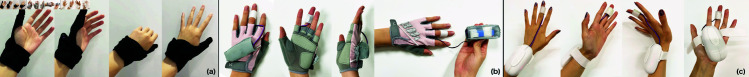
The wearable design iteration of the heart rate sensor. (a) a wearable band fixes the PPG sensor on the thumb. (b) a glove design with electronic components on the back of the hand. (c) a 3D-printed case encapsulating electronic components and being worn on the wrist.

As shown in Fig 3, to achieve unobtrusive and comfortable heart rate sensing, the wearable design adopted the form of a hand-worn garment. It consists of a PPG sensor, an Arduino board, a Bluetooth module, and a 3.7V, 1000 mAh rechargeable lithium battery. The PPG sensor connects to an Arduino board which processes the raw pulse signal into a series of Inter-Beat-Interval (IBI) data. The IBI data are then wirelessly sent to the PC program via a Bluetooth connection. The first design prototype (Fig 3a), which fixes the PPG sensor on the thumb, resulted in more noise in the pulse signal due to hand movements. In the user test, it was observed that the second prototype depicted in Fig 3b may lead to sweaty palms for pregnant women during prolonged use. In our final iteration (Fig 3c), the PPG sensor is covered with an adjustable ring-shaped structure that can be worn to various sizes of fingers for comfort and aesthetic considerations. The Arduino board, Bluetooth module, and battery are embedded in a 3D-printed case worn on the wrist. The case is easy to clean and keeps hygienic.

**Fig 4:**
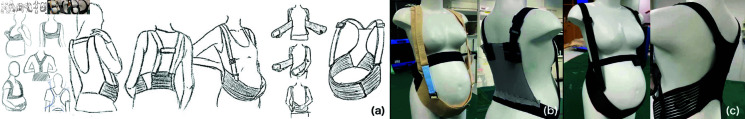
The wearable design iteration of the breathing sensor. (a) the ideation process of an adjustable maternity belt. (b) a jacket-shape maternity belt prototype for the convenience of wearing. (c) the fabric stretch sensor module is easily taken off for charging replacement.

For respiration sensing, GlowGrow integrated a flexible fabric sensor from Elastech (www.elas-tech.com) into a maternity belt, which is easy and comfortable to wear by pregnant women alone (Fig 4). This wearable garment could help fix the breathing sensor on the user’s abdomen position. To achieve optimal accuracy and robustness, we explored various methods to vertically and horizontally embed the fabric sensor into different positions on the maternity belt. It is determined that the position around the abdomen, below the breasts is the most suitable for pregnant women performing breathing exercises, as depicted in Fig 4c. When the user wears the breathing-sensing maternity belt, the expansion and contraction of the abdomen and chest cavity during breathing induce plastic deformation in the fabric stretch sensor. This deformation is transformed into changes in electrical data, captured by the circuit module, and transmitted to the computer via a Bluetooth connection.

### Ambient user interface

In the GlowGrow system, we leverage ambient light and nature soundscape as an ambient user interface for Biofeedback and Bio-feedforward information display. Compared to graphic user interfaces (GUIs), ambient interfaces place minimal constraints on users and thus offer a more comfortable condition for relaxation training. On the other hand, as an environmental stimulant, colored light can also provoke emotional and physiological responses, such as reducing an individual’s physiological arousal level and improving subjective feelings of calmness [90, 91]. Similarly, nature sounds and sedative music with a slow tempo and low tones can bring comfortable and relaxed feelings and are proven to affect blood pressure for primiparous postpartum women [77, 92].

Specifically, regarding the lighting interface, the GlowGrow system consists of three ambient light sources: a ceiling lamp, a floor lamp, and a portable lamp. The light parameters including brightness, saturation, and hue can be controlled via the PC program in real time. Regarding the audio interface, GlowGrow employed a synthesized nature soundscape (NS) based on the NS model proposed in [93]. The constructed soundscape consists of wind, river, and various bird sounds. The NS model offers multiple parameters that could control the perceptual quietness and richness of the soundscape. In the following sections, we elaborate on the mapping from heart rate variability and respiration data to the parameters of ambient light and nature sound-scape for Biofeedback display.

### Biofeedback and bio-feedforward design

In the GlowGrow system, two different working modes: Biofeedback and Bio-feedforward, were developed to assist pregnant women in deep breathing relaxation. In Biofeedback mode, users receive information about their breathing and heart rate variability (HRV). The biofeedback information helps them regulate breathing patterns in real-time to achieve a higher HRV level. In Bio-feedforward mode, users receive a standard 0.1Hz slow-paced breathing guidance. They practice deep breathing by following the guidance.

#### Biofeedback mode.

The GlowGrow Biofeedback mode integrates two types of biofeedback systems: heart rate variability (HRV) biofeedback and respiration biofeedback. HRV Biofeedback is a widely used technique for stress management and relaxation training. It is considered an effective biomarker of stress, with prolonged decreased HRV associated with mental stress [94]. Because of respiratory sinus arrhythmia (RSA), the heart rate increases during inhalation and decreases during exhalation. This rhythmic variation in heart rate, known as short-term HRV, is linked to the respiratory cycle. Consequently, short-term HRV [95] is extensively utilized in biofeedback to guide individuals in controlling and enhancing their heart rate variability, particularly through deep breathing practice [96]. Compared to HRV Biofeedback, the working of the respiration biofeedback system is relatively straightforward. The user’s breathing pattern is continuously monitored and promptly provided as feedback, allowing them to be aware of their breathing and learn to control the duration and depth of inhalation, exhalation, as well as the duration of pauses after inhalation and exhalation [97].

**Fig 5:**
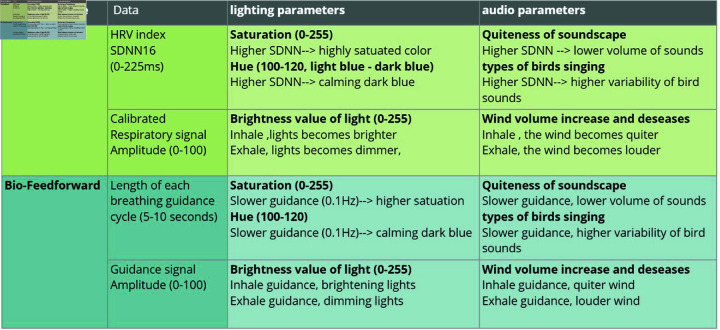
Mapping from biofeedback and bio-feedforward information to the control parameters of ambient light and nature soundscape.

To implement this dual-biofeedback in our system, heart rate variability (HRV) and respiration data are measured using a PPG sensor in a wearable garment and a fabric stretch sensor in a maternity belt. The processed HRV and respiration data are mapped to the control parameters of ambient light and the nature soundscape as shown in Fig 5. Through the changing environment, pregnant women can intuitively receive feedback information and become more aware of their HRV level and respiratory pattern. Then learn to regulate their breathing patterns, and prac-tice slow-paced deep breathing to improve the relaxation effect, which is reflected by the raised HRV.

**Mapping the HRV data to the saturation and the hue of ambient light, and to the ‘richness’ and ‘quietness’ of nature soundscape.**

In this study, we use a wearable photoplethysmograph (PPG) sensor to measure the blood volume pulse (BVP) signal from the user’s finger [98]. The raw BVP signal first gets processed using a peak detection algorithm into interbeat interval (IBI) data. Then, the standard deviation of IBI (SDNN) is calculated with a moving window of 16 heartbeats as a time-domain HRV index. This time window (W=16) is large enough to include at least one complete respiratory circle and small enough to be sensitive to changes in breathing pattern [99]. The *SDNN*_16_ is calculated by the following equation as the HRV Biofeedback information in the GlowGrow system.

$$IBI_{avg}=\frac{15\times IBI_{avg}+IBI}{16}$$ (1)

$$SDNN_{16}=\frac{15\times SDNN_{16}+|IBI-IBI_{avg}|}{16}$$ (2)

The calculated *SDNN*_16_ are mapped to two lighting parameters: the saturation and hue of light. When users perform deep breathing smoothly and achieve an optimal relaxation status, their RSA is enhanced, and the HRV, *SDNN*_16_ value, is increased, which is represented by the more highly saturated ambient light and a soundscape with a quieter but richer variety of nature sounds. The detailed mapping is shown in Fig 5.

**Mapping the breathing signal to the brightness of lighting, and to the wind sound in nature soundscape.**

Based on the real-time breathing signal, the measured respiratory amplitude is mapped to the brightness of the light. The exhalation process makes the light dim and the inhalation process makes the light brighter, respectively. In the nature soundscape, the respiratory amplitude mainly controls the volume of wind sounds. When the user breathes in, the sound of the wind becomes smaller and when the user breathes out, the wind becomes louder, respectively. The mapping from the breathing signal to the GlowGrow interface is shown in Fig 5.

#### Bio-feedforward mode.

In the Bio-feedforward mode, the GlowGrow system provides users with pre-set, fixed-pace guidance at the resonant frequency of 0.1 Hz, which is proven to increase cardiac oscillations and enhance physiological relaxation [100, 101]. This breathing guidance is presented through the regular changes in the ambient interface of GlowGrow. The light and the nature soundscape differentiated for inhalation and exhalation. Specifically, the increasing light brightness and wind volume instruct users to inhale until the light starts to dim and the wind turns quiet, which indicates that the users need to exhale. The pregnant women were requested to synchronize breathing with the guiding light and soundscape.

**Fig 6:**
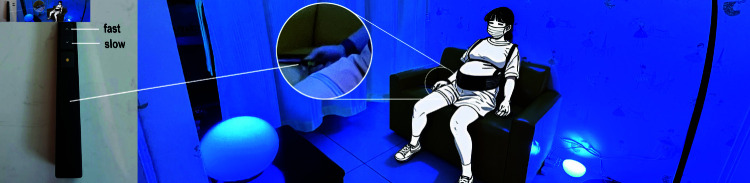
The remote controller is conducted to adjust the guidance frequency in Bio-feedforward session.

Most traditional Bio-feedforward systems [102] use fixed breathing guidance throughout the training session. However, as suggested by [102], deep breathing exercises with prolonged inhale and exhale might cause breathing fatigue. Therefore, to avoid breathing fatigue, GlowGrow allows users to adjust the initial guidance frequency during the session by using a remote controller, as shown in Fig 6. The Bio-feedforward session starts with a standard 0.1 Hz breathing guidance and pregnant women can shorten the guidance when they feel tired or difficult to follow. Also, they can prolong the guidance again.

## Evaluation

### Participants

The evaluation was conducted between June 12, 2023, and September 20, 2023. A total of 24 women in their late-stage pregnancies participated in this study. Their average age was 29.125±3.6 (range: 20-35, M=29.125, SD=3.6), while their average gestational age was 35.2±3.2. We established a scientific research collaboration with the hospital and the user study was approved by the Affiliated People’s Hospital of Ningbo University. The user study was conducted following the hospital’s guidelines and regulations, with informed written consent obtained from all participants. The individual in this manuscript has given written informed consent (as outlined in PLOS consent form) to publish these case details.

All pregnant women were recruited using the snowball sampling method from the antenatal clinic of a local hospital while they performed their regular prenatal care checks. Those who have complications during pregnancy, such as pregnancy-induced hypertension, gestational diabetes, cardiac or psychiatric disorders, and other high-risk pregnancy conditions, were excluded from this study. Furthermore, participants did not receive any medical HRV or respiration Biofeedback training.

### Experimental setup and procedures

**Fig 7:**
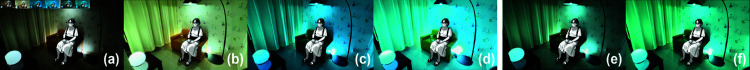
Women in their late pregnancy experiencing the Biofeedback (a to d) and Bio-feedforward (e, f) modes of the system. (a) Breath out, low HRV (b) Breath in, low HRV (c) Breath out, high HRV (d) Breath in, high HRV (e) Breath out lead by the feedforward lights (f) Breath in lead by the feedforward lights.

GlowGrow system has been deployed in a lactation room next to the antenatal clinic. To create a controlled environment for our experiment, we partitioned the room using a curtain, separating the space into two sections. One section was used to operate the system, while the other was designated for the participants to perform relaxation training. A sofa was placed in the middle of this space with four lights surrounding it. Two Philips Hue (www.developers.meethue.com) were placed on the ground side by the sofa. A ball light was put on the desk in front of the sofa. And a standing lamp was used to provide overhead light from above. The light from different sources constructs an ambient lighting environment for relaxation training (Fig 7).

**Fig 8:**
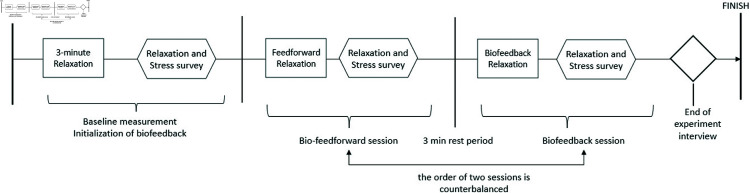
The experimental procedure.

As shown in Fig 8, the experiment consisted of four phases: (1) introduction, (2) baseline measurement, (3) and (4) Biofeedback or Bio-feedforward relaxation session. In *phase 1*, participants were informed about the experiment’s procedures and were required to sign a consent form. Subsequently, they sat on a couch and were fitted with a fingertip PPG sensor and an abdomen breathing sensor. In *phase 2*, participants relaxed for 3 minutes with a regular breathing pattern, during which we collected the baseline of their heart rate variability and respiration data for later data analysis. After 3-minute relaxation, participants also completed questionnaires to measure their subjective stress baseline. Additionally, the range of HRV and respiration amplitude was calculated to initialize the Biofeedback program for GlowGrow.

In *phase 3* and *phase 4*, the pregnant woman performed two sessions of deep-breathing relaxation with either Biofeedback or the Bio-feedforward mode of GlowGrow. Between two relaxation sessions, the participant rested for 3 min. The experiment followed a within-subject design with counterbalancing to avoid carry-over effects. For each session, we collected the participant’s physiological data and the self-reports on stress and relaxation via the questionnaires. At the end of the experiment, a follow-up interview was conducted to collect qualitative data which helped us to understand user experience with the GlowGrow system.

### Biofeedback and bio-feedforward protocol

In two relaxation sessions *(phase 3 & 4)*, the participants were exposed to the same lighting environment and natural soundscape. They were suggested to relax with deep breathing. With the Bio-feedforward mode of GlowGrow, we suggested the participants try to perform deep breathing by following the guidance represented by the changing light and wind sounds. When they feel tired with the guidance, they could use the remote controller to adjust the length of guidance to breathe comfortably. With the Biofeedback mode, the participants were informed that the volume of wind sound and brightness of light would increase and decrease with their breathing and when they performed well in the relaxation exercise, the soundscape would become quiet and simple and the light would become dark blue color.

### Data collection and data analysis

In the study, we collected both psychological and physiological data to evaluate the effectiveness of GlowGrow in facilitating relaxation for pregnant women. To measure participants’ self-reported levels of relaxation and anxiety, we employed the State-Trait Anxiety Inventory (STAI) [43] and the Relaxation Rating Scale (RRS) [44]. Additionally, physiological stress parameters such as heart rate (HR), the standard deviation of R-R intervals (SDNN), low-frequency to high-frequency heart rate variability ratio (LF/HF), and respiration rate were measured using the embedded photoplethysmography (PPG) sensor and breath sensor, respectively. Prior to the experiment, we asked participating pregnant women to complete the Pregnancy Stress Rating Scale [45] to assess their overall level of stress. We then conducted semi-structured interviews with the participants after the experiment to gather information on their experience with both the Biofeedback and Bio-feedforward modes of the GlowGrow system. The interview questions are listed in Table 1. All participants consented to have their interviews recorded, and the data collected were transcribed for analysis.

**Table 1:** The semi-structured interview questions.

Theme	Interview Questions
Biofeedback Mode	Can you feel the lighting and music change with your heart rate and respiration in this session? Can you describe the change? Does it make you relaxed?
	You control the lighting brightness by respiration, is that helpful for your breathing? How and why?
Bio-feedforward Mode	Can you feel the lighting and music changing at a specific rate in this session? Does it make you relaxed?
	Is the fixed changing lighting and music helpful for your breathing? How and why?
General Questions	Can you describe the differences you experienced between the two sessions?
	Can you describe what you like and what you dislike experiencing about the system?
	What other scenarios do you think the system can be deployed to in the future?
	What do you think of deploying such wearable devices to collect your heart rate and breathing data during your pregnancy?

## Results

### Quantitative results

Since we had a small sample size, a Shapiro-Wilk test was first conducted to test the normality of STAI, RRS, HR, SDNN, breathing rate, and LF/HF data under the three different conditions (baseline, Biofeedback, and Bio-feedforward). The results show that HR and SDNN data satisfy a normal assumption: HR (*HR*_*baseline*_(*W* = 0.958,*p* = 0.396), *HR*_*biofeedback*_(*W* = 0.987,*p* = 0.984), *HR*_*biofeedforward*_(*W* = 0.982,*p* = 0.933)) and SDNN (*SDNN*_*base*_(*W* = 0.937,*p* = 0.138), *SDNN*_*biofeedback*_(*W* = 0.928,*p* = 0.087), *SDNN*_*biofeedforward*_(*W* = 0.949,*p* = 0.254)). Therefore, one-way repeated-measures ANOVA was conducted to compare the HR and SDNN between the three conditions. Friedman Test was conducted to examine the effect of Biofeedback and Bio-feedforward on STAI, RRS, breathing rate, and LF/HF data as these data do not satisfy normal distributions according to Shapiro-Wilk tests.

#### GlowGrow enhances HRV in relaxation.

**Fig 9:**
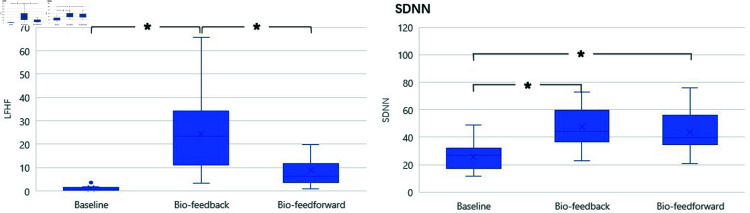
The results of Heart Rate Variability: SDNN and LF/HF.

Fig 9 shows the results of two HRV indexes: SDNN and LF\HF. There is a significant difference in SDNN among the three conditions. (F(2,46)=35.335,p<0.0005). Post hoc analysis with a Bonferroni adjustment revealed that SDNN was statistically significantly higher in the Biofeedback condition M=47.6,SD=17.07,p<0.005 and the Bio-feedforward condition M=43,8,SD=14,38,p<0.005 than in baseline M=25.5,SD=8.86. There is no significant difference between Biofeedback and Bio-feedforward conditions*p* = 0.313. Regarding the LF\HF, there was a statistically significant difference in LF\HF among three conditions x2(2)=31.083,p=0.000. The LF\HF in the Biofeedback condition M=24.4,SD=15.69 was significantly increased compared to the baseline condition M=1.1,SD=1.105,p=0.000 and the Bio-feedforward condition M=8.9,SD=7.6,p=0.000. Regarding the heart rate (HR), there is no significant difference (F(2,46)=6.077,P=0.005) among the three conditions: baseline M=89.4,SD=10.36(beatsperminute,bpm), Biofeedback M=86.5,SD=10.31(bpm), Bio-feedforward M=86.5,SD=10.18(bpm).

#### GlowGrow facilitates deep breathing.

**Fig 10:**
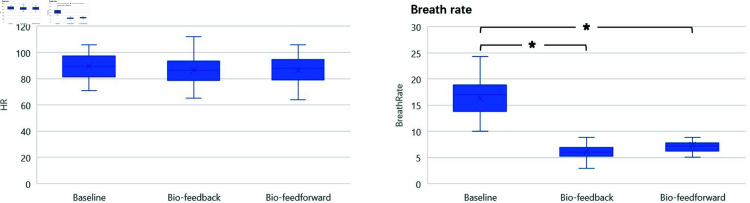
The results of Heart rate and breathing rate.

As shown in Fig 10, Biofeedback and Bio-feedforward interventions led to statistically significant differences in respiration rate, *X*^2^(2) = 39,*p* = 0.000. The median respiration rates for the baseline, Biofeedback, and Bio-feedforward were 17 (13.83 to 18.92), 6.06 (5.25 to 6.91) and 7.19 (6.28 to 7.84) (cycle per minute, cpm), respectively. The respiration rate was significantly reduced in Biofeedback condition (Z=−4.286,p=0.000) and Bio-feedforward condition (Z=−4.286,p=0.000) compared to baseline condition. Besides, the respiration rate in the Biofeedback condition is also significantly lower than in the Bio-feedforward condition (Z=−2.616,p=0.009).

#### GlowGrow reduces subjective stress.

The results of the Pregnancy Stress Rating Scale (PSRC) show that more than half of the participating pregnant women reported a moderate level of stress in their late pregnancy. And 3 out of 24 pregnant women experienced relatively high stress. The main reasons causing pregnancy stress are fetal safety, labor pain, delivery safety, accidents during delivery, and fetal health conditions.

**Fig 11:**
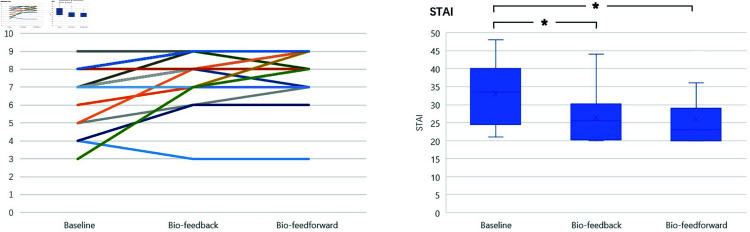
The results of Relaxation Scale and STAI stress survey.

Pregnant women’s subjective stress was measured by STAI and RRS. The results are shown in Fig 11. According to the Friedman Test, there was a statistically significant difference in RRS depending on the Biofeedback and Bio-feedforward interventions, *X*^2^(2) = 20.780,*p* = 0.000. The median of perceived relaxation levels for the baseline, Biofeedback, and Bio-feedforward conditions were 7 (5.25 to 8), 8 (7 to 9), and 8.5 (8 to 9), respectively. There was a statistically significant increase in perceived relaxation in the Biofeedback (Z=−3.328,p=0.001) and Bio-feedforward (Z=−3.342,p=0.001) compared to baseline. However, there were no significant differences in perceived relaxation between Biofeedback and Bio-feedforward (Z=−1.732,p=0.083).

Also, the Biofeedback and Bio-feedforward interventions led to a statistically significant difference in STAI self-reported stress and anxiety level, *X*^2^(2) = 24.427,*p* = 0.000. Median perceived stress levels for the baseline, Biofeedback, and Bio-feedforward were 33.5 (24.5 to 40), 25.5 (20.25 to 30.25) and 23 (20 to 29), respectively. There was a statistically significant reduction in perceived stress levels in Biofeedback (Z=−4.147,p=0.000) and Bio-feedforward session (Z=−3.747,p=0.000) compared to baseline. However, there were no significant differences in stress levels between Biofeedback and Bio-feedforward (Z=−0.928,p=0.354).

### Qualitative results

#### GlowGrow can help pregnant women cope with stress.

Most women (19 out of 24) stated the GlowGrow system can be helpful during their pregnancy. 8 women stated that they would like to use the system to cope with extreme moods (e.g., moody, depressed, angry, tired). P1 stated *“It helps me to observe my body’s status, which sets my mind at rest”.* P5 said *“Pregnancy made people anxious. I’m a nurse so I may know more about anxiety generation. The system can help mothers understand their anxiety through this device and be able to take control”.* P20 said *“... the understanding of our psychology and cognition is lacking. It is valuable if we have some diagnosis to refer to”.* P24 said *“It is extremely important to practice deep breathing skills because breathing can reduce a lot of pain during delivery. As for how to breathe, we don’t know as we have never given birth. With this equipment, I think it allows us to better regulate my breath ”.* Moreover, P22 consider the system shows humanistic care, *“I think it is very important to monitor the mood for mothers during their pregnancy, which reflects humanistic care”.*

#### Both modes of the GlowGrow system facilitate deep breathing in relaxation.

From the interview, most pregnant women (22/24 for Bio-feedforward, and 21/24 for Biofeedback, respectively) stated the GlowGrow system could help them with deep breathing exercises for relaxation. For instance, P15 said that *“It *Biofeedbackmode* helped me regulate my breathing pattern because when I make the lights dim, I try to breathe out deeply to make it turn off completely. And when I make it brighten, I also try to breathe in deeply to make it shining”.* However, 4 women (P1, P3, P8, P9) claimed that the Bio-feedforward mode was a bit challenging for them to follow. For example, P9 said that *“I need to think about the lighting in my mind all the time, like when it is shining, I need to take a breath in. Sometimes I felt I left behind and I can’t follow it very well”.* P20 admitted that she found it difficult to follow the guidance at the beginning, but then she got more concentrated during the second session.

#### GlowGrow helps pregnant women better relaxed.

Most pregnant women agreed that the environment created by the GlowGrow system with ambient light and soundscape could make them feel relaxed. Specifically, 19 out of 24 said that the Bio-feedforward mode made them relaxed, and 23 out of 24 stated that the Biofeedback mode made them relaxed. 3 women felt their baby’s movement during deep breathing (P1, P9, P20) and indicated that the deep breathing helped them release their pain and discomfort. P1 said that *“I felt he (or she) moved a lot during my breath. (he or she) seems very active. Breathe with the lights made my belly feel better”.* P20 stated *“I felt my body clearly. And I felt my baby’s movement. It made me relaxed. Actually, I think it was a transition of people’s attention. The music and lighting made me feel comfortable and prodigious which is hard to expressive”.* P3 experienced uterine contractions during the breathing exercises, she indicated the relaxing environment is helpful for pain relief. *“Because I was about to give birth, I was having uterine contractions in the middle of the training process. I think it’s good that the contractions were not so strong. It’s good. I think it’s good to have it (the GlowGrow system)”.*

#### Biofeedback is more popular than bio-feedforward.

In the interview, we asked pregnant women to make a comparison between the Biofeedback and Bio-feedforward modes. Most of the participants preferred Biofeedback mode over Bio-feedforward because it allows users to control the environment instead of requiring efforts to follow the Bio-feedforward guidance. 13/24 pregnant women (P1, P3, P5, P6, P8, P9, P10, P13, P14, P17, P19, P22, P24) stated that Biofeedback mode took less effort to practice deep breathing. On the contrary, they argued that following the lighting guidance in Bio-feedforward mode requires effort and focus, and it was easy to get tired when their breath rate could not match the guidance. For instance, P9 thinks Biofeedback mode is *“more casual”*. She said she doesn’t have to *“look for patterns of the lighting and pay attention to follow its behavior”*. 11/24 women (P2, P6, P9, P10, P11, P12, P14, P17, P18, P22, P24) claimed that Biofeedback mode is a responsive mode giving the capability of controlling the environment by regulating their breathing. For example, P6 said that *“The first one (Biofeedback) is more autonomous. I feel more comfortable inhaling and exhaling with my own rhythm”.* P10 stated that *“The first one (Biofeedback) is more pleasing to control the light by myself. I have the autonomy”.*

#### Bio-feedforward requires user more effort to match breathing guidace.

Five women (P1, P5, P7, P18, P22) had difficulty with Bio-feedforward mode. They stated that the dimming and brightening of the lights could clearly represent the breathing guidance, but they found it difficult to adjust their breathing to match the pace of guidance. For instance, 3 of them (P1, P18, P22) felt the dimming process was too quick for them to fully exhale. P18 said *“While it (Bio-feedforward) shows inhale, I feel like it was a long time, but suddenly it’s dimming within a very short time. I can not follow its pace. It took me a few rounds before I adjust myself to its pace”.* However, some (P15, P23) claimed that the Bio-feedforward mode could help them practice deep breathing more efficiently than the Biofeedback mode. P15 said that *“I prefer the first one (Bio-feedforward) because I felt if I lead the light, I can’t control the rhythm very well. So let the lights lead me would be better”.* Similarly, P23 said that *“... I feel better following it because my breath can last longer”.*

#### Customized guidance and interfaces to improve user experience.

17% participants mentioned that they hope to see more possibilities for customization in the future GlowGrow system. Specifically, 4 women (P2, P3, P7, P16) suggested the pace and pattern of Bio-feedforward guidance can be customized according to an individual’s physiological capacity for deep breathing. For instance, P7 indicated that *“The exhale process can be slower and the darkening process (of the light guidance) can be a little longer. I feel that the inhale is perfect, but the exhale can be longer. Because I just finished exhaling all the air, and can not catch my breath to follow the glowing light immediately”.* Furthermore, some participants mentioned ’personalized’ features for GlowGrow. They want the system could measure and store the guidance breathing patterns with which they achieve the best relaxation results. For example, P2 suggested *“The main improvement is that I want its first breath following my own breath. It (my previous breath pattern) is recorded, and it can be carried over to the next time I practice breathing exercises”.*

Regarding the light interface, 3 women (P2, P8, P9) mentioned that they hoped the color of the lighting could be customized so that they could set the color of their favorite ones, such as yellow or pink. For instance, P9 said *“...blue is calming. But yellow would be better for me. I feel that in a dark environment like this, warm colors would be more comfortable to look at. Probably the eyes do not get tired easily”.* Regarding the audio interface, P20 indicated that she likes the audio interface with nature sounds and relaxing music, and the lighting feedback might be an optional interface to use. *“I like the music and lights quite much. The rhythm made me feel comfortable. I prefer to close my eyes, just listen to the music, and follow the wind sound. I open my eyes occasionally (to see the lights)”.*

#### GlowGrow for home, workplaces, and psychological consultations.

Besides the antenatal clinic setting in this study, 6 participants (P1, P5, P9, P21, P10, P20) suggested the GlowGrow system is also suitable for a comforting environment like home. For instance, P20 stated that *“I believe this music and light system (GlwoGrow) can make my home very comfortable. So it might be a good system for me or other people who want to try deep breathing exercises at home alone to relax and deal with some negative thoughts I have in mind ”.* 5 women (P3, P5, P11, P18, P22) mentioned using this system in the workplace. P22 felt the stress from her work during her pragnancy *“To be honest my work is more stressful...I still work in the company, this system might help me with my stress at the workplace”.* Besides, 4 women (P2, P6, P16, P24) also mentioned the system being used in psychological consultations. Last but not least, P16 emphasized the importance of deploying GlowGrow system for pregnant women to relieve prenatal pain, *“when there is a paroxysm, it is not possible to go pain-free before the opening of 1 to 3 fingers. So it is necessary to relieve the pain by some external means. So music and deep breathing can be a distraction to relieve pain and eliminate tension. Well, other surgeries may use anesthesia, then there is no point. So in this situation the pain can hardly be avoided, it (the system) can bring soothing effects”.*

## Discussion

### The benefits of Biofeedback breathing training worth further exploration in fetal heart rate, natural delivery rate, and birth outcomes

Feeling stressed and anxious is a common phenomenon among pregnant women. Decreased HRV has been found caused by anxiety in previous research [103]. Our field study focused on the benefits of training pregnant women to learn deep breathing and relaxation techniques prior to delivery. Doctors and nursing facilitators in our study emphasized the importance of relaxation in ensuring a smooth and comfortable delivery experience for the mother. The doctors believe that the more relaxed the mother is, the smoother the delivery process will be. This is supported by literature that highlights the benefits of deep breathing, including improved oxygenation and pain relief [104–106]. Fear and anxiety will produce muscle tension, which leads to the increased perception of pain for pregnant women [107], which obstacle to the childbirth process. Our study suggests that Biofeedback-assisted deep breathing and relaxation can help to reduce physiological stress and promote relaxation during pregnancy.

Such Biofeedback interventions can increase pregnant women’s HRV and reduce their breathing rate. In the long term, using Biofeedback regularly during the whole pregnancy may also help them improve their relaxation skills, better manage stress, and further promote their health and well-being, which is also beneficial for their newborns [108]. Our user study with the GlowGrow system showed that some women felt a stronger connection with their babies and experienced their movements during breathing exercises. Additionally, the fetal heart rate monitoring test after the user study showed improved clarity and performance according to doctors. Further research is recommended to explore the advantages of regular Biofeedback interventions in areas such as fetal heart rate, natural childbirth rates, and birth outcomes.

### GlowGrow should be simple to set up, easy to use, and better fit the pregnant women’s routine

In this study, we designed the GlowGrow system to facilitate immersive relaxation training. It transforms a traditional biofeedback system into a physiology-driven relaxation environment via newly developed wearable bio-sensors and ambient information displays. Based on the interview results, we found that the users need a simpler setup and an easier way to operate the system. we found the current system can be iterated further in terms of the preparation work to get started with the training. As one of the participants mentioned, she would probably not use the GlowGrow system frequently at home because it takes too much time for her to set it up. But when she was waiting for the antenatal appointments, with support from the nurse, she enjoyed using the system for relaxation training. Some participants suggested that the procedure needs to be simplified. For instance, One participant said *“she first needs to find a relatively dark environment, then wear the wearables, turn on and connect all the lights, and finally relax”.* The long time preparation would be a barrier for pregnant women to use it at home. Besides, some also mentioned they don’t have time during the day for training. Arranging the training at night (e.g., before sleep) would be more convenient for them. Therefore, the Biofeedback intervention should be settled properly into pregnant women’s context and should be flexible enough to fit into their routines.

### The feedback modality can be customized according to pregnant women’s cognitive perception

560 Researchers have recommended the use of multimodal displays in information systems to reduce cognitive workload [109]. In the context of GlowGrow, where Bio-feedforward guidance or Biofeedback information is conveyed to users, a combination of visual and audio modalities is used, as they complement each other. Similar findings are found in the RESonance system [22]. However, we also found a few participants prefer to use an unimodal information display. One woman stated that either the lighting interface or the nature soundscape provided enough information for her. Also, two women indicated they preferred audio display, especially when they would like to relax with their eyes closed. The light could be an optional interface for them. Therefore, we suggest that the future design of the biofeedback relaxation system allows users to select and personalize the feedback modality based on their preferences and context. Finding a way to design biofeedback that effectively conveys information and facilitates a relaxation experience, without causing cognitive burden, is a significant design challenge for future work.

## Conclusion

In this paper, we introduce GlowGrow, an innovative Biofeedback and Bio-feedforward system designed to assist expectant mothers in practicing deep breathing and relaxation exercises leading up to childbirth. GlowGrow leverages wearable designs and ambient user interface of lights and nature soundscape to present Biofeedback data and Bio-feedforward guidance. Through our user study, we found that both modes of GlowGrow can effectively reduce physiological and psychological stress levels, slow breathing, and induce a state of relaxation among pregnant women. Our research suggests that the use of Biofeedback and Bio-feedforward intervention during pregnancy and childbirth has the potential to enhance natural delivery outcomes and promote healthier birth experiences for both the mother and child.

Future studies may explore the broader benefits of biofeedback-assisted breathing training for pregnant women, including its impact on physiological and psychological outcomes, such as improved fetal heart rate regulation, higher natural delivery rates, and better overall birth outcomes. Additionally, as consistent use of biofeedback throughout pregnancy may help women develop effective relaxation techniques and manage stress more efficiently, the long-term effects of using biofeedvack on regular basis could be further investigated. Finally, improving the "ease of use" and adaptability of ambient biofeedback systems like GlowGrow to seamlessly integrate into users’ routines is a valuable area for exploration. Simplifying the setup process and enabling users to personalize feedback modalities could enhance engagement and accessibility. Further design research is needed to provide effective relaxation guidance with minimal cognitive effort, ensuring these systems are practical, user-friendly, and supportive across diverse contexts.
